# Supplementary material for: HIVprotI: an integrated web based platform for prediction and design of HIV proteins inhibitors
Source: J Cheminform. 2018 Mar 9;10:12. doi: 10.1186/s13321-018-0266-y (PMC5845081; doi:10.1186/s13321-018-0266-y)
Supplement: Supplementary file 2 — Additional file 2. Source code of HIVProtI web server. [file 13321_2018_266_MOESM2_ESM.zip › HIVprotI_Source-code/hivprotI/weka-3-6-6/doc/weka/gui/experiment/package-tree.html]

weka.gui.experiment Class Hierarchy


---


|  |  |  |
| --- | --- | --- |
| **Overview** \| **Package** \| Class \| **Tree** \| **Deprecated** \| **Index** \| **Help** \| **Weka's home** | |  |
| **PREV**   **NEXT** | **FRAMES**    **NO FRAMES**     **All Classes** |  |


---


## Hierarchy For Package weka.gui.experiment

**Package Hierarchies:**: All Packages

---

## Class Hierarchy

- java.lang.Object
  - java.awt.Component (implements java.awt.image.ImageObserver, java.awt.MenuContainer, java.io.Serializable)
    - java.awt.Container
      - javax.swing.JComponent (implements java.io.Serializable)
        - javax.swing.JLabel (implements javax.accessibility.Accessible, javax.swing.SwingConstants)
          - javax.swing.DefaultListCellRenderer (implements javax.swing.ListCellRenderer, java.io.Serializable)
            - weka.gui.experiment.**AlgorithmListPanel.ObjectCellRenderer**
          - javax.swing.JPanel (implements javax.accessibility.Accessible)
            - weka.gui.experiment.**AlgorithmListPanel** (implements java.awt.event.ActionListener)- weka.gui.experiment.**DatasetListPanel** (implements java.awt.event.ActionListener)- weka.gui.experiment.**DistributeExperimentPanel**- weka.gui.experiment.**Experimenter**- weka.gui.experiment.**GeneratorPropertyIteratorPanel** (implements java.awt.event.ActionListener)- weka.gui.experiment.**HostListPanel** (implements java.awt.event.ActionListener)- weka.gui.experiment.**ResultsPanel**- weka.gui.experiment.**RunNumberPanel**- weka.gui.experiment.**RunPanel** (implements java.awt.event.ActionListener)- weka.gui.experiment.**SetupModePanel**- weka.gui.experiment.**SetupPanel**- weka.gui.experiment.**SimpleSetupPanel**
        - java.awt.Window (implements javax.accessibility.Accessible)
          - java.awt.Dialog
            - javax.swing.JDialog (implements javax.accessibility.Accessible, javax.swing.RootPaneContainer, javax.swing.WindowConstants)
              - weka.gui.experiment.**OutputFormatDialog**
    - weka.gui.experiment.**ExperimenterDefaults** (implements java.io.Serializable)- weka.gui.experiment.**Messages**

---


|  |  |  |
| --- | --- | --- |
| **Overview** \| **Package** \| Class \| **Tree** \| **Deprecated** \| **Index** \| **Help** \| **Weka's home** | |  |
| **PREV**   **NEXT** | **FRAMES**    **NO FRAMES**     **All Classes** |  |


---
